# Reconstruction of Molecular Interaction Patterns from Endolysosomes in Ceramide-Depleted Cells

**DOI:** 10.1021/acs.nanolett.6c01272

**Published:** 2026-05-04

**Authors:** Yiqing Feng, Florian Gärber, Cecilia Spedalieri, Stephan Werner, Christoph Pratsch, Christoph Arenz, Stephan Seifert, Janina Kneipp

**Affiliations:** † Department of Chemistry, 9373 Humboldt-Universität zu Berlin, Brook-Taylor-Str. 2, 12489 Berlin, Germany; ‡ Einstein Center of Catalysis (EC2/BIG-NSE), 14915 Technische Universität Berlin, Marchstr. 6-8, 10587 Berlin, Germany; § Hamburg School of Food Science, Department of Chemistry, Universität Hamburg, Grindelallee 117, 20146 Hamburg, Germany; ∥ 28340 Helmholtz-Zentrum Berlin für Materialien und Energie GmbH, Department X-ray Microscopy, Albert-Einstein-Str. 15, 12489 Berlin, Germany

**Keywords:** ceramide synthase (CerS), surface enhanced Raman scattering (SERS), Fumonisin B1, gold nanoparticles, surrogate minimal depth (SMD), ceramide

## Abstract

Ceramide synthase (CerS) is a key enzyme in sphingolipid metabolism, predominantly maintaining lipid homeostasis within the endoplasmic reticulum (ER). Capturing the biochemical consequences of CerS activity at the subcellular level is of great interest to understand complex lipid flux in living cells. Here, surface-enhanced Raman scattering (SERS) combined with soft X-ray tomography and random forest (RF) machine learning was employed to probe the cellular response to CerS inhibition. The nanospectroscopy data from endolysosomes and changes in ultrastructure demonstrate diverging alterations upon the sequential administration of CerS inhibitor Fumonisin B1 and SERS probe gold nanoparticles. RF analysis reveals distinct spectral patterns of the affected endolysosomes, characteristic of a ceramide imbalance under different conditions, indicating a tight metabolic coupling between the ER and endolysosomes. The vibrational nanospectroscopy coupled with computational reconstruction of molecular interaction patterns and ultrastructural imaging provides a comprehensive strategy to understand enzyme activity and metabolic dysregulation in situ.

## Graphical abstract

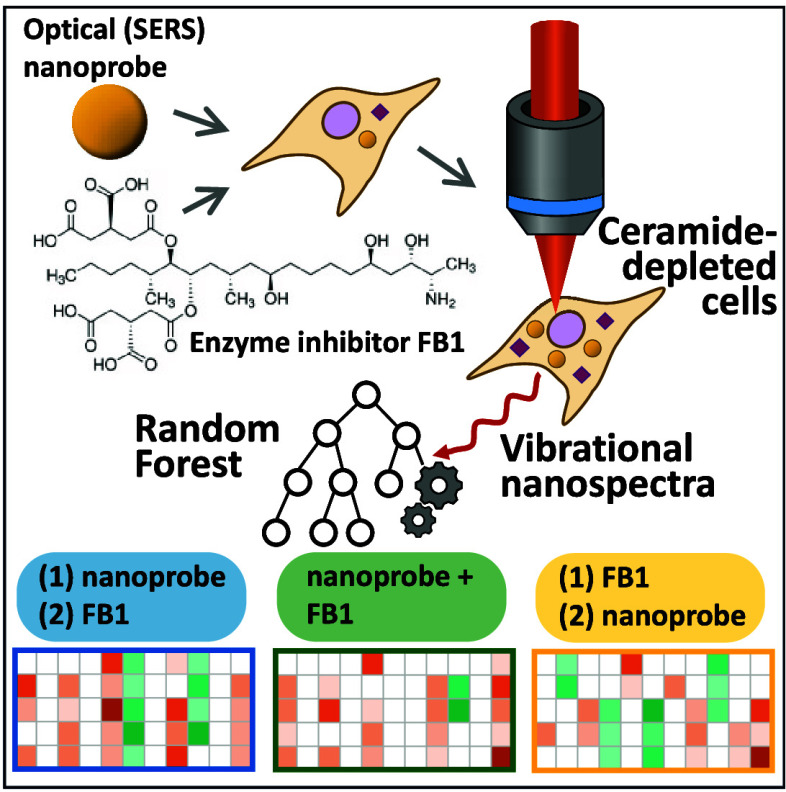

Ceramide synthase (CerS) serves as a key enzyme in ceramide metabolism.^1^ In the endoplasmic reticulum (ER), CerS catalyzes the generation of dihydroceramide, the ceramide precursor, from acyl-CoA and sphinganine through a de novo pathway.^2–4^ Alternatively, CerS can also reutilize long-chain sphingosine through reacylation in a salvage pathway to form ceramide.^5,6^

Fumonisin B1 (FB1) disturbs ceramide synthesis by nonselectively inhibiting all six CerS isoenzymes.^7^ As a secondary metabolite from Fusarium verticillioides and Fusarium proliferatum, it is prevalently found in contaminated cereal-related food stock.^8,9^ With a structure similar to sphingoid bases (Scheme 1), FB1 competitively inhibits CerS and blocks dihydroceramide generation.^8,10^ CerS inhibition by FB1 has been shown to disrupt lipid homeostasis, thereby activating autophagy that is mediated by ER stress^11^ and inducing toxicity driven by oxidative stress.^12^ Due to the tight functional and physical connections between intracellular organelles, the impacted sphingolipid metabolism in the ER is expected to alter the endolysosomal environment and to affect its lipid composition and functions.^13,14^ Such connections have been suggested by molecular changes in endolysosomes that are observed upon excess uptake of fluorescently labeled ceramide into the Golgi.^15,16^

**Scheme 1:**
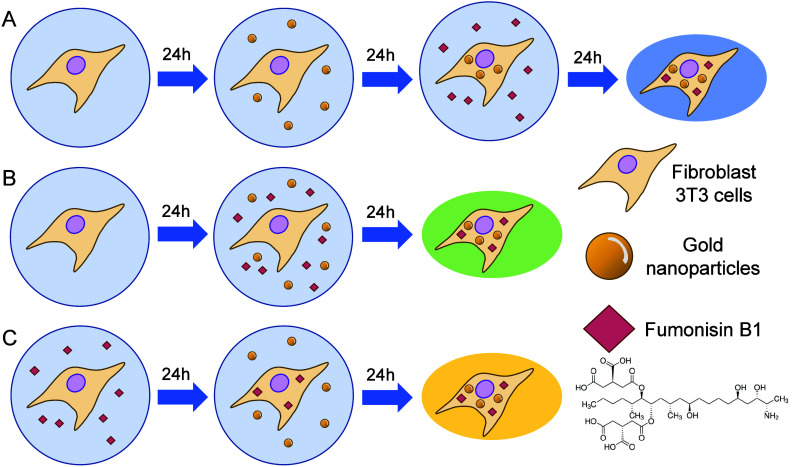
Incubation Schemes for the Cell Samples Incubated Gold Nanoparticles (as SERS Nanoprobes) and the CerS Inhibitor FB1^a^

a 3T3 cells were incubated with gold nanoparticles (**A**) prior to 24h incubation with 10 μM FB1, (**B**) together with 10 μM FB1 for 24h, and (**C**) after seeding and growing with 10 μM FB1 for 24h.

Here, we describe the endolysosomal response to the FB1-induced inhibition of CerS at the level of protein and lipid structure and interaction. In the experiment, 3T3 fibroblast cells were incubated with FB1 and gold nanoparticles. The latter are included in endolysosomes that are monitored by highly localized molecular probing using surface-enhanced Raman scattering (SERS).^17–20^ A varied incubation sequence (Scheme 1) enables variation of the onset of endolysosome formation with respect to the onset of inhibition: (i) exposure to 10 μM FB1 for 24 h following a 24 h incubation with gold nanoparticles to inhibit CerS after formation of probed endolysosomes (Scheme 1A), (ii) simultaneous CerS inhibition and formation of the endolysosomes (Scheme 1B) through the administration of 10 μM FB1 and gold nanoparticle at the same time for 24 h, and (iii) seeding and growing cells in 10 μM FB1 for 24 h prior to 24 h incubation with gold nanoparticles to include the optical nanoprobes into endolysosomes that form in a ceramide-depleted environment (Scheme 1C). In all cases, the inhibition of the enzyme continues in the ER while SERS probing takes place in the endolysosomes.

The molecular information is inferred from the vibrational signals that arise from the immediate environment of the intra-endolysosomal gold nanoparticles. The spectral data obtained from cells with CerS inhibition (Figure 1) and corresponding control samples (Figure S1) show the typical interactions of internalized gold nanoparticles with proteins and lipids of the endolysosomes (cf. Table S1).^15,21,22^ The signals that are visible in average spectra (Figure 1A–C) and from an analysis of relative occurrence of respective bands in the data sets of thousands of spectra (Figure 1D–F) are assigned to vibrations of specific functional groups, elements of secondary structure, and lipid structure and composition that change when CerS is inhibited (see detailed comparisons of Figure 1 and Figure S1 in the Supporting Information).

**Figure 1:**
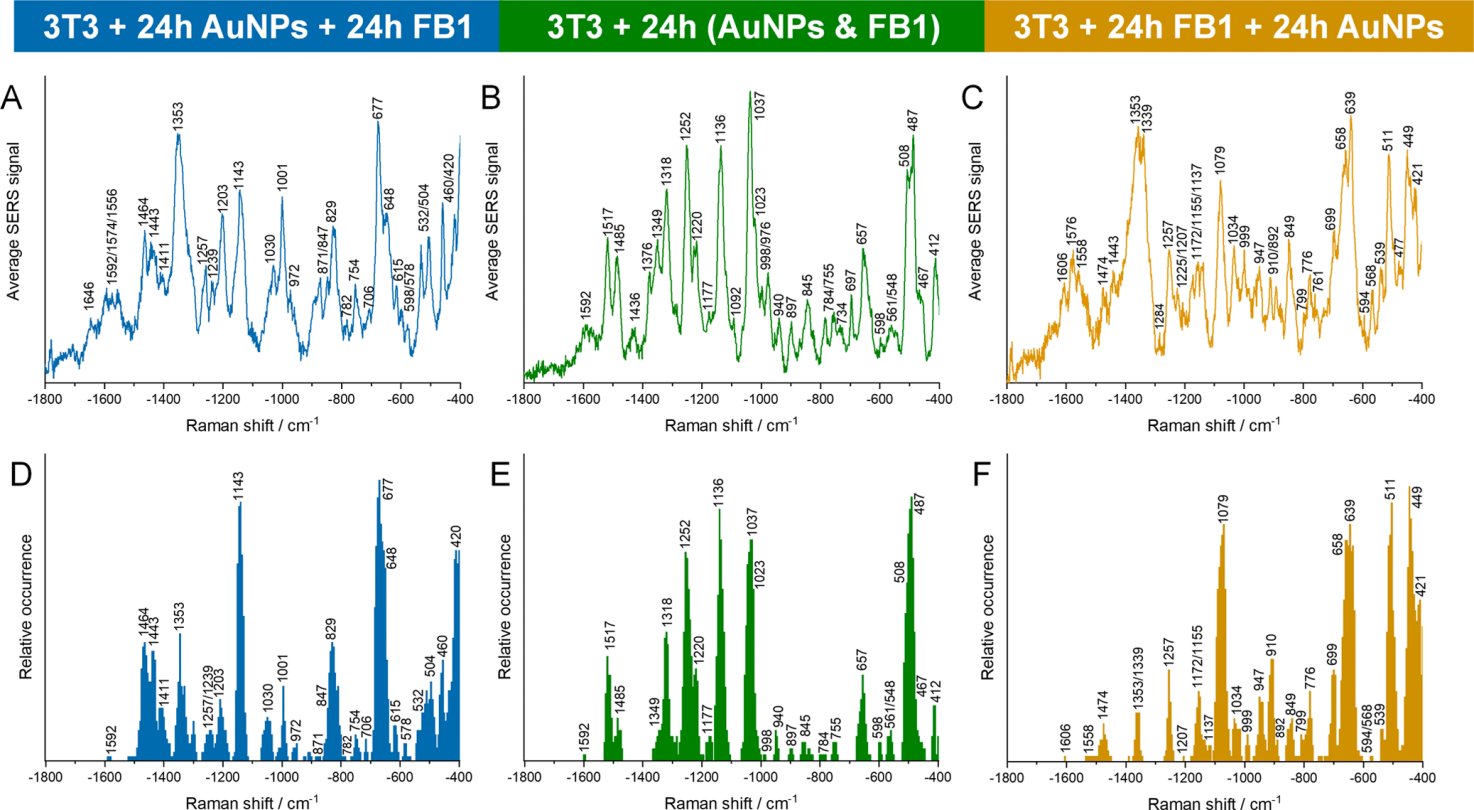
**(A, B, C)** Average SERS spectra (vector-normalized) and **(D, E, F)** relative band occurrence in the respective data sets of 3T3 cells incubated with gold nanoparticles for 24h **(A, D)** prior to FB1 following Scheme 1A, **(B, E)** together with FB1 following Scheme 1B, **(C, F)** after seeding and growing with FB1 for 24h following Scheme 1C. The data sets contain 172 (A, D), 104 (B, E), and 342 (C, F) SERS spectra, respectively, after elimination of spectra with no signal. Excitation wavelength: 785 nm. Excitation intensity: 2.7 × 10^5^ W cm^–2^. Acquisition time: 1 s.

The combination of vibrational signals in endolysosomes that are already present during CerS inhibition (Scheme 1A) varies from those found in endolysosomes that form during the inhibitor administration (Scheme 1B) and in cells where the de novo synthesis of ceramide has been inhibited for 24 h (Scheme 1C, Figure 1, and Table S1). The loadings (eigenspectra) of principal component analyses (PCA) of spectra from inhibitor-treated cells and their controls reveal distinct changes when endolysosomes form at different time points relative to CerS inhibition (Figure S2 and the discussion thereof). Specifically, the endolysosomal molecular structure and composition show a more drastic response to decreased ceramide when FB1 administration (and CerS inhibition in the ER) occurs before or simultaneously with the formation of the endolysosomal membranes. The partial group separation, where a few spectra from treated cells show greater differences to the control groups, especially along the first principal component (PC1, Figure S2A,S2D,S2G), indicates that the endolysosomal environment in some subsets of measured cells might be more sensitive to CerS dysfunction in the ER than others.

Nevertheless, only a more detailed assessment of signal co-occurrence can inform about a combined presence of different molecules in the femtoliter volumes that are probed in the SERS approach. We further developed here a spectral analysis based on random forest-surrogate minimal depth (RF-SMD).^23^ This approach was demonstrated to be very robust toward well-known challenges posed by the strong signal fluctuations that are inherent to SERS, especially at low molecule concentration and when high enhancement regimes are used.^22,24^ More importantly, the representation of variable relation applying the mean adjusted agreement (MAA) in a spectrally resolved fashion can deconstruct the complex responses to the level of co-occurrence of functional groups and structural elements^15,22,25^ and, therefore, identify specific local molecular environments of proteins and lipids.

When CerS inhibition occurs after the formation of probed endolysosomes (Scheme 1A), most of the important bands (Figure 2A, vertical axis, obtained from Figure S3) co-occur with a network of signals that include the lipid C–C stretching at 1101 cm^–1^,^26,27^ the phosphate group O–P–O stretching at 798 cm^–1^,^22^ amide III components at 1288 cm^–1^,^28^ 1277 cm^–1^,^21,29^ and ∼ 1240 cm^–1^,^22,29^ and the S–S bond at 475 cm^–1^ ^30,31^ (Figure 2A, bold labels above horizontal axis). This indicates that major observed molecular changes due to FB1 treatment involve changes of the protein secondary structure near lipids of the endolysosomal membrane. Different from the important amide III signal at 1288 cm^–1^,^28^ another amide III component at 1277 cm^–1^ ^21^, ^29^ exhibits fewer related variables, without, e.g., the bands of proline/valine at 977 cm^–1^ ^21^ or lipid phosphate heads at 798 cm^–1^ ^22^ (Figure 2A, top row with the star). The presence of these two kinds of protein–lipid interactions implies that the conformation of proteins varies in very localized microdomains. The importance and mutual relation of bands of phenylalanine side chains at 1002 cm^–1^ ^29,32^ and disulfide at 679 cm^–1^ ^30,31^ point to partial unfolding in the proteins. Moreover, the signal of phosphatidylinositol at 787 cm^–1^ ^29,33^ (Figure 2A, green arrow), one of the key signaling molecules, co-occurs with tryptophan (1347 cm^–1^)^22,30^ and phenylalanine (1002 cm^–1^)^29,32^ bands. This indicates that a known endolysosomal signaling process associated with repair mechanisms^34^ must have been affected indirectly as well.

**Figure 2:**
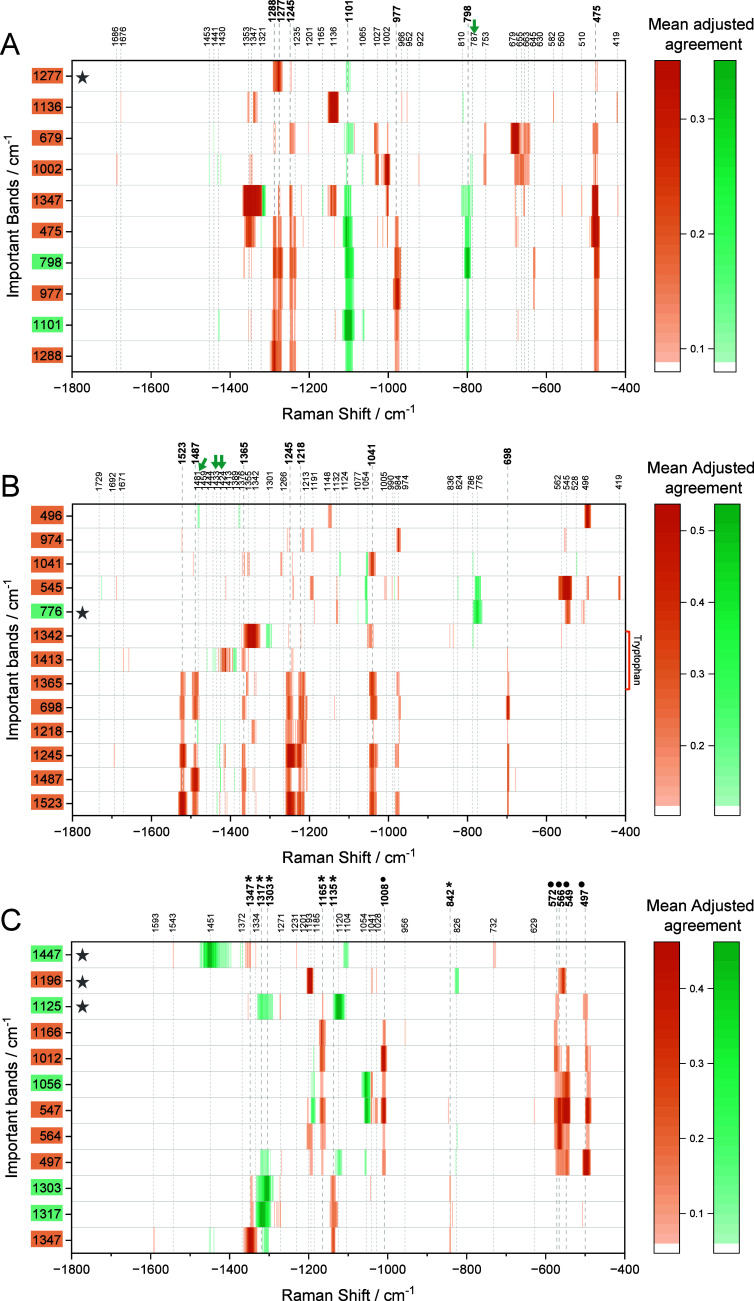
Spectral variables co-occurring with the important bands (vertical axis, selected according to Figure S3) obtained by RF-SMD of SERS spectra from cells incubated with endolysosomal nanoprobes for 24h **(A)** prior to FB1, **(B)** together with FB1, **(C)** after seeding and growing with FB1 for 24h and their respective control group. Signals representing proteins and lipids are shown in orange and green blocks, respectively. Band assignments are shown in Table S1. Asterisk and black dot symbols on the horizontal axis highlight distinct groups of colocalized lipid and protein signals. Green arrows highlight narrow signals that are discussed specifically.

A different co-occurrence pattern was observed in endolysosomes that formed during exposure to FB1 (Figure 2B). It is composed of amide II components at 1523 cm^–1^ ^33^ and 1487 cm^–1^,^30^ amide III components at 1245 cm^–1^ and 1218 cm^–1^,^29,33^ a tryptophan vibration at 1365 cm^–1^,^29^ and C–S signals at 698 cm^–1^ ^33^ (Figure 2B, bold labels). The predominant changes in protein structures are illustrated, e.g., by three different tryptophan signals at 1365 cm^–1^,^29^ 1342 cm^–1^,^22,32^ and 1413 cm^–1^ ^35^ that displayed different localized environment through their association with different bands, including some lipid signals, likely pointing to diverse orientation and structure of membrane proteins upon CerS inhibition. A few distinct interactions with lipids were observed through the different combinations of lipid methylene deformation bands, e.g., at 1424 cm^–1^,^21,30,36^ 1433 cm^–1^,^29,33^ and 1481 cm^–1^,^30^ (Figure 2B, green arrows), correlated with important amide II and III components. They suggest that proteins undergoing varying conformational changes may locate near membranes of heterogeneously packed structures. An important signal of phosphatidylinositol at 776 cm^–1^ ^29,33^ (Figure 2B, row with a star) co-occurs with protein vibrations that indicate significant changes in protein structure, e.g., the backbone vibration at 1132 cm^–1^,^21,31^ an amino acid signal at1191 cm^–1^,^29,30^ bands of disulfide groups at 545 cm^–1^ ^30,37^ and 496 cm^–1^,^21,31,38^ and with lipid intrachain C–C stretching bands at 1077 cm^–1^ and 1054 cm^–1^.^26,27,37^ These changes in the lipid chain state-of-order suggest significant conformational rearrangements of colocalized membrane proteins when the endolysosomal membrane forms in the presence of FB1.

When cells are grown in the presence of the inhibitor before the nanoprobe incorporation into the endolysomes, the importance of lipid signals in differentiating treated from control cells and molecular colocalization with lipids increases (Figure 2C), indicating that the formation of the endolysosomal membranes during CerS inhibition in the ER induces significant molecular alterations that localize to different positions or domains in these membranes. For example, the lipid chain packing marker at 1447 cm^–1^ ^21,22,29^ (Figure 2C, top row with the star) is associated with amide signals at 1543 cm^–1^ ^21,29^ and 1231 cm^–1^,^21,22,29^ tryptophan vibrations at 1347 cm^–1^ ^22,30^ and 732 cm^–1^,^33^ a lipid tail deformation mode at 1372 cm^–1^,^22^ and an intrachain stretching band at 1104 cm^–1^.^26,27^ This indicates that parts of the membranes undergo changes in rigidity and nearby protein structures are altered. Another lipid–protein co-occurrence pattern is observed for important bands of tryptophan at 1347 cm^–1^,^22,30^ lipid CH_2_/CH_3_ deformation at 1317 cm^–1^,^15,21^ and the twisting mode at 1303 cm^–1^ ^33,39^ that show relation with each other and with the protein C–C/C–N bond at 1135 cm^–1^ ^21,31^ and the tyrosine ring breathing mode at 842 cm^–1^ ^32^ (Figure 2C, all bold labels with asterisk). A further correlation pattern, indicating more diverse molecular interactions, was found for important bands at 1056 cm^–1^, 1012 cm^–1^, 564 cm^–1^, 547 cm^–1^, and 497 cm^–1^ that are assigned to lipid asymmetric C–C stretching,^26,27,37^ phenylalanine,^29^ tryptophan,^29^ and disulfide S–S modes,^21,31,38^ respectively (Figure 2C, all bold labels with dots). In addition to the important lipid signal assigned to an asymmetric C–C stretching mode at 1056 cm^–1^,^26,27^ the corresponding symmetric mode at 1125 cm^–1^ ^26,27^ (Figure 2C, row with a star) forms a separate pattern in association with different variables, including signals of protein backbone at 1165 cm^–1^,^29,30^ amide III at 1271 cm^–1^,^21,29^ tryptophan at 1347 cm^–1^,^22,30^ and other lipid signals assigned to chain deformation modes at 1303 cm^–1^ ^33,39^ and 1317 cm^–1^.^15,21^ These observations collectively imply distinct localizations of altered proteins on the membrane regions of different structures. Moreover, the phospholipid PO_2_ stretching signal at 826 cm^–1^ ^29,40^ alongside the signals of tyrosine at 1041 cm^–1^ ^22^ and phenylalanine at 1028 cm^–1^ ^32,37,41^ co-occurred with a band of aromatic amino acids at 1196 cm^–1^ ^29,30^ (Figure 2C, the row with a star). This points to a changed exposure of protein side chains to the surface of phospholipid membranes.

The results show that the exposure to the CerS inhibitor provokes different dynamic cellular changes depending on the SERS probing with respect to the onset of the metabolic disruption. The altered molecular interactions found in the vibrational spectra indicate a response of the cell as a whole to sphingolipid imbalance and reflect the tight metabolic coupling between ER and endolysosomes. In agreement with the involvement of different organelles in such a response, the cellular ultrastructure, visualized by soft X-ray tomography (SXT, Figure 3), shows modifications when CerS is inhibited. When the onset of CerS inhibition by exposure to FB1 was not timed after or before the delivery of the SERS nanoprobes (Figure 3A and 3C, following Scheme 1A and 1C, respectively) but in coexposure with the nanoprobes (following Scheme 1B), swollen mitochondria with increased cristae spacing were evident (Figure 3B). This points to a mitochondrial fission-fusion imbalance,^42^ likely due to the cells coping with FB1 action in the ER and simultaneously engaging in active endocytosis of the gold nanoparticles and therefore failing to overcome the multitude of stressors in the ER induced by the disrupted ceramide homeostasis. Correspondingly, the predominant protein contributions to the spectral differences (Figures 1B,E and 2B) could be an indicator that the dysfunctional response in the endolysosomes is triggered by this combined stress. The observed impact on mitochondria ultrastructure agrees with reports that FB1 treatment in SH-SY5Y neuroblastoma cells resulted in an accumulation of reactive oxygen species in mitochondria and therefore mitochondrial depolarization,^43^ which can substantially increase mitochondrial volume.^44^ A 3D nanotomographic reconstruction of the ER, the FB1-targeted compartment, proved to be very challenging and was not achieved here. The gold nanoparticles and loose nanoparticle agglomerates of different sizes are observed inside vesicular, endolysosomal structures (Figure 3A-C, yellow arrowheads), corresponding to our previous findings on endocytotic processes of gold nanoparticles in 3T3 cells.^45^ Comparing the size distribution with that in the controls (Figure S4), initial exposure to FB1 (following Scheme 1C) led to a significant change (*P* < 0.5) in the overall distribution profile, with more individual nanoparticles and/or small agglomerates (Figure 3D). The observed alterations of cellular ultrastructure and nanoprobe distribution corroborate an influence of the lack of ceramide in the ER on endolysosomal function, in agreement with the variability at the molecular level.

**Figure 3:**
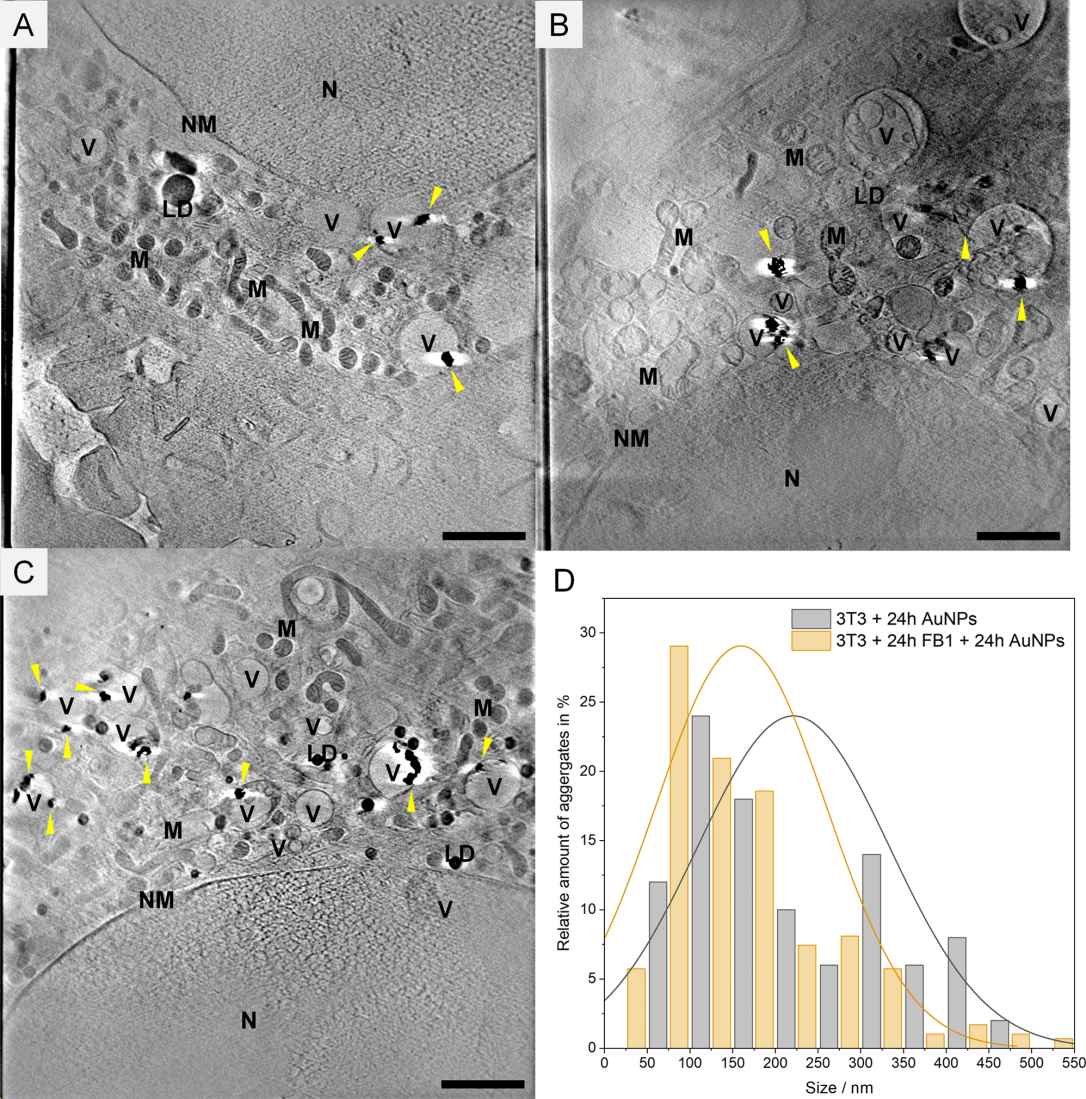
Results of SXT nanotomography. Tomographic slices of 3T3 cells incubated with gold nanoparticles for 24h **(A)** prior to FB1, **(B)** together with FB1, **(C)** after seeding and growing with FB1 for 24h. **(D)** Distribution of the relative number of intracellular aggregates of a given approximate size based on the SXT data from 3T3 cells incubated with AuNPs for 24h (gray, control groups) and 3T3 cells incubated with gold nanoparticles for 24h after seeding and growing with FB1 for 24h (yellow, following Scheme 1C). Gold nanostructures are marked with yellow arrowheads. Scale bar: 2 μm. M: mitochondrion, V: vesicle, NM: nuclear membrane, N: nucleus, LD: lipid droplets.

In conclusion, ceramide reduction in the ER and its effects on sphingolipid metabolism were observed to have significant downstream impacts on the endolysosomal environment. The observation of ultrastructural modifications in the endolysosomal system itself but also in other compartments, together with alterations of molecular structure and interaction, as well as signaling pathways, underpin the connection between the ER and the endolysosome in the cellular response to metabolic stress. This is, moreover, supported by the dependence of the altered endolysosomal protein–lipid interactions on the onset of lipid metabolic disruption. The reconstruction of specific molecular interaction patterns by machine learning analysis of vibrational information and 3D ultrastructural data shows great potential to probe cellular reactions induced by a disrupted lipid metabolism. It will facilitate our understanding of enzyme activity and lipid trafficking at the subcellular level in future work.

## Supplementary Material



## Notes

SERS spectra of endolysosomes in 3T3 fibroblast cells undergoing inhibition of CerS by Fumonisin B1 (FB1) differ from those of control cells; Principal component analysis of SERS spectra of the endolysosomes in 3T3 fibroblast cells undergoing inhibition of CerS by FB1; Variable importance analysis by surrogate minimal depth from random forest models of SERS data sets of the endolysosomes in 3T3 cells undergoing inhibition of CerS by FB1; Ultrastructure of 3T3 cells incubated with gold nanoparticles as control samples; Methods (PDF)

## Abbreviations

CerS: ceramide synthase
ER: endoplasmic reticulum
FB1: Fumonisin B1
PCA: principal component analysis
RF: random forest
SERS: surface-enhanced Raman scattering
SMD: surrogate minimal depth
SXT: soft X-ray tomography.
