# Supplementary material for: Reconstruction of Molecular Interaction Patterns from Endolysosomes in Ceramide-Depleted Cells
Source: Nano Lett. 2026 May 4;26(18):6174–81. doi: 10.1021/acs.nanolett.6c01272 (PMC13178127; doi:10.1021/acs.nanolett.6c01272)
Supplement: Supplementary file 1 [file nl6c01272_si_001.pdf]

Supporting Information

Reconstruction of Molecular Interaction Patterns from Endolysosomes in Ceramide Depleted Cells

Yiqing Feng[‡#], *Florian Gärber*[§], *Cecilia Spedalieri*[‡], *Stephan Werner*[ʬ], *Christoph Pratsch*[ʬ], *Christoph Arenz*[‡], *Stephan Seifert*[§], *Janina Kneipp*[‡*]

[‡] Department of Chemistry, Humboldt-Universität zu Berlin, Brook-Taylor-Str. 2, 12489 Berlin, Germany

[#] Einstein Center of Catalysis (EC2/BIG-NSE), Technische Universität Berlin, Marchstr. 6-8, 10587 Berlin, Germany

[§] Hamburg School of Food Science, Department of Chemistry, Universität Hamburg, Grindelallee 117, 20146 Hamburg, Germany

[ʬ] Helmholtz-Zentrum Berlin für Materialien und Energie GmbH, Department X-ray Microscopy, Albert-Einstein-Str. 15, 12489 Berlin, Germany

Table of contents

SERS spectra of endolysosomes in 3T3 fibroblast cells undergoing inhibition of CerS by Fumonisin B1 (FB1) differ from those of control cells S1
Principal component analysis of SERS spectra of the endolysosomes in 3T3 fibroblast cells undergoing inhibition of CerS by FB1 .... S5
Variable importance analysis by surrogate minimal depth from random forest models of SERS data sets of the endolysosomes in 3T3 cells undergoing inhibition of CerS by FB1 .... S8
Ultrastructure of 3T3 cells incubated with gold nanoparticles as control samples S10
Methods S11
References S14

SERS spectra of endolysosomes in 3T3 fibroblast cells undergoing inhibition of CerS by Fumonisin B1 (FB1) differ from those of control cells

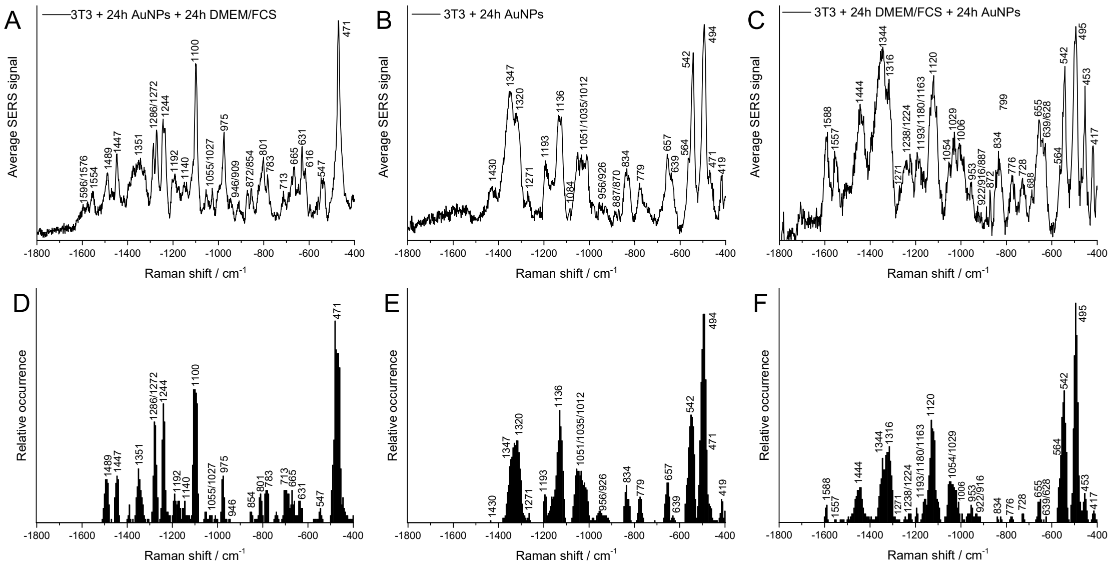

Figure S1. (A, B, C) Average SERS spectra and **(D, E, F)** relative band occurrence of the respective SERS data sets of **(A, D)** 3T3 cells incubated with gold nanoparticles for 24h prior to 24h incubation with the fresh medium as the control samples for Scheme 1A in the main text, **(B, E)** 3T3 cells incubated with gold nanoparticles for 24h as the control samples for Scheme 1B in the main text, **(C, F)** 3T3 cells incubated with gold nanoparticles for 24h after seeding and growing in the fresh medium for 24h as the control samples for Scheme 1C in the main text. The data sets contain 177 (A, D), 105 (B, E), and 155 (C, F) SERS spectra, respectively, after elimination of spectra with no signal after the experiment. Excitation wavelength: 785 nm. Excitation intensity: 2.7×10^5 W cm^{-2}. Acquisition time: 1 s

The differences between the spectral data of cells where CerS was inhibited by FB1 were found to differ for the three types of incubation experiment (illustrated in Scheme 1 of the main text).

When CerS is inhibited after the exposure to the SERS nanoprobes (Scheme 1A in the main text), the amide I signal appears at 1646 cm^{-1},[1] and the amide III components at 1286 cm^{-1} and 1272 cm^{-1}[2-4] are missing (Figure 1A), while the presence of the 1143 cm^{-1} band attributed to protein backbone stretching [1, 3, 5] increase pronouncedly (compare Figure 1D of the main text and Figure S1D). These features indicate an exposure of the protein backbone due to altered secondary structures. The frequently occurring CH_2 deformation modes of lipids at 1464 cm^{-1}[1, 4, 6-7] and 1443 cm^{-1}[3-6] (Figure 1A and 1D), in the absence of signals that indicate acyl chain conformation at 1100 cm^{-1}[8-9] and 1055 cm^{-1}[8-10] or a phospholipid band at 801 cm^{-1}[6] (compare Figure 1A and 1D with Figure S1A and S1D), point toward a heterogenous structure of the endolysosomal membranes in cells that respond to the lack in

ceramide. Additional signals of phosphatidylinositol and cholesterol at 598 cm^{-1}[4] and 532 cm^{-1},[6] respectively, (compare main text Figure 1A with Figure S1A) reflect a re-organization of membrane components.

When the fibroblast cells were exposed to SERS nanoprobes and the inhibitor simultaneously (Scheme 1B in the main text), other spectral differences were observed between the spectra of the treated cells and control samples (compare main text Figure 1B and Figure S1B). Protein structural changes become evident by the presence of amide II signals at 1517 cm^{-1}[7] and 1485 cm^{-1},[1] and amide III components at 1252 cm^{-1} and 1220 cm^{-1}[4, 7] as well as the C–C/C–N vibration at 1136 cm^{-1} of the protein backbone [3, 5] that have a more frequent occurrence in the treated cells (Figure 1B and 1E in the main text, Figure S1B and S1E, Table S1). Additional bands assigned to phenylalanine at 1023 cm^{-1}[10-12] and 998 cm^{-1},[4, 12] and tryptophan ring breathing modes at 755 cm^{-1} and 734 cm^{-1}[1, 4-5] (Figure 1B) suggest an increased exposure of hydrophobic amino acid residues. A C-S stretching mode of methionine at 697 cm^{-1}[7] (main text Figure 1B) and a less frequently occurring disulfide band at 548 cm^{-1}[1, 10] (main text Figure 1E) indicate that some proteins undergo a local destabilization. Further, the spectra from cells where CerS was inhibited present a symmetric intra-chain vibration of lipids at 1092 cm^{-1}[8-9] (main text Figure 1B), different from the spectra from the control group that display asymmetric modes at 1084 cm^{-1} and 1051 cm^{-1}[8-10] (Figure S1B), suggesting the changes in membrane state-of-order. The phospholipid and phosphatidylinositol signals at 1177 cm^{-1}[3, 6, 13] and 598 cm^{-1}[4] (main text Figure 1B), respectively, indicate an altered membrane composition compared to the control spectra.

Differently, when the cells were exposed to FB1 prior to gold nanoparticle incubation (Scheme 1C in the main text), so that CerS inhibition is ongoing when the endolysosomes form the spectra of the cells treated with the inhibitor (main text Figure 1C) have more bands in common with the controls (Figure S1C, cf. Table S1). A few protein-related differences are observed, including the presence of an amide II signal at 1576 cm^{-1} together with an amide III component at 1257 cm^{-1}[4, 7] (main text Figure 1C), additional bands assigned to aromatic amino acid residues at 1606 cm^{-1},[4, 12, 14] 1172 cm^{-1},[3-4, 10, 12] and 1034 cm^{-1}.[6] These modes replace the signals of aromatic side chains that are found in the control spectra at 1029 cm^{-1}[10-12] and 628 cm^{-1}[4, 7, 15] (compare Figure 1C in the main text and Figure S1C). A frequent occurrence of the C-S and S-S bands at 658 cm^{-1}[3, 5-6, 16] and 449 cm^{-1},[1, 5] respectively, (main text Figure 1F) and the shift of the S-S band to 511 cm^{-1}[3, 5, 16] (main text Figure 1C) compared to the control spectra (Figure S1F and S1C, respectively) could result from a local destabilization of proteins that accompany new protein-protein interactions upon CerS inhibition. Further, a re-organization of lipids is revealed by the frequent occurrence of signals assigned to a C–C stretching mode at 1079 cm^{-}

[18-9] (main text Figure 1C and 1F), as well as a band of lipid phosphate groups at 799 cm^{-1} [6] (main text Figure 1C), and a more frequent signal of the CH_2 scissoring mode at 1474 cm^{-1} [1] (main text Figure 1F). Also here changes of membrane composition include an increased presence of phosphatidylinositol, as indicated by the band at 594 cm^{-1} [4] (main text Figure 1C).

Table S1. Raman shifts and tentative assignments of bands in the SERS spectra of 3T3 fibroblast cells of all samples in this study. Assignments based on Ref [1, 3-7, 9-12, 15-22].

Raman Shift / cm^{-1}	Tentative assignment	Raman Shift / cm^{-1}	Tentative assignment
1724	Lipid ester group	1027	Phe C-H ip bend
1671	Amide I	1000	Phe R br
1646	Amide I	984	Lipid =CH bend
1606	Tyr, Phe R str, Amide I	974	Pro, Val C–C str
1591	C=C str, COO^- str	951	Pro, Val C–C str
1576	Amide II, C–C str,COO^- str	923	Pro, Val C–C str
1556	Amide II, Trp, Tyr, COO^- str	912	Pro C–C str
1542	Amide II	890	Pro, Val C–C str
1517	Amide II	871	Pro, Val C–C str
1500	Amide II	849	Tyr R br
1487	Amide II	832	Tyr R br
1475	Lipid CH_2 def	824	Phospholipid PO_2 str
1464	Lipid CH_2 def	801	Lipid O–P–O str
1444	Lipid CH_2 def	776	Phosphatidylinositol
1433	Lipid CH_2 def	766	Trp R def
1411	Trp COO^- str	755	Trp R br
1388	Lipid CH_3 bend	737	Trp R br
1376	Lipid CH_3 def	725	DNA, A
1367	Trp	706	Cholesterol R def
1349	Trp	698	C–S str
1339	Trp	679	C–S str
1318	Lipid CH_2/CH_3 def	659	C–S str
1303	Lipid CH_2 def	636	Tyr C–C twist
1286	Amide III	628	Phe C–C twist
1274	Amide III	618	cholesterol
1255	Amide III	597	Phosphatidylinositol
1240	Amide III	578	S-S Str
1220	Amide III (β sheet structure)	564	Trp
1198	Tyr, Trp, Phe	545	S-S Str
1178	Lipid phosphate ester	532	Cholesterol
1171	Tyr C–H bend	522	S–S str
1160	Protein C–N/C–C str	503	S–S str
1137	Protein C–N/C–C str	489	S–S str
1120	Lipid C–C sy str	473	C–S str
1100	Lipid C–C sy str	454	S–S str
1078	Lipid C–C as str	431	Cholesterol
1053	Lipid C–C as str	417	Trp
1037	Tyr R def		

Abbreviations: Str, stretching; def, deformation; twist, twisting; br, breathing; bend, bending; ip, in-plane; R, ring; Trp, tryptophan; Tyr, tyrosine; Phe, phenylalanine; Pro, proline; Val, valine; A, adenine; sy, symmetric; as, antisymmetric

Principal component analysis of SERS spectra of the endolysosomes in 3T3 fibroblast cells undergoing inhibition of CerS by FB1

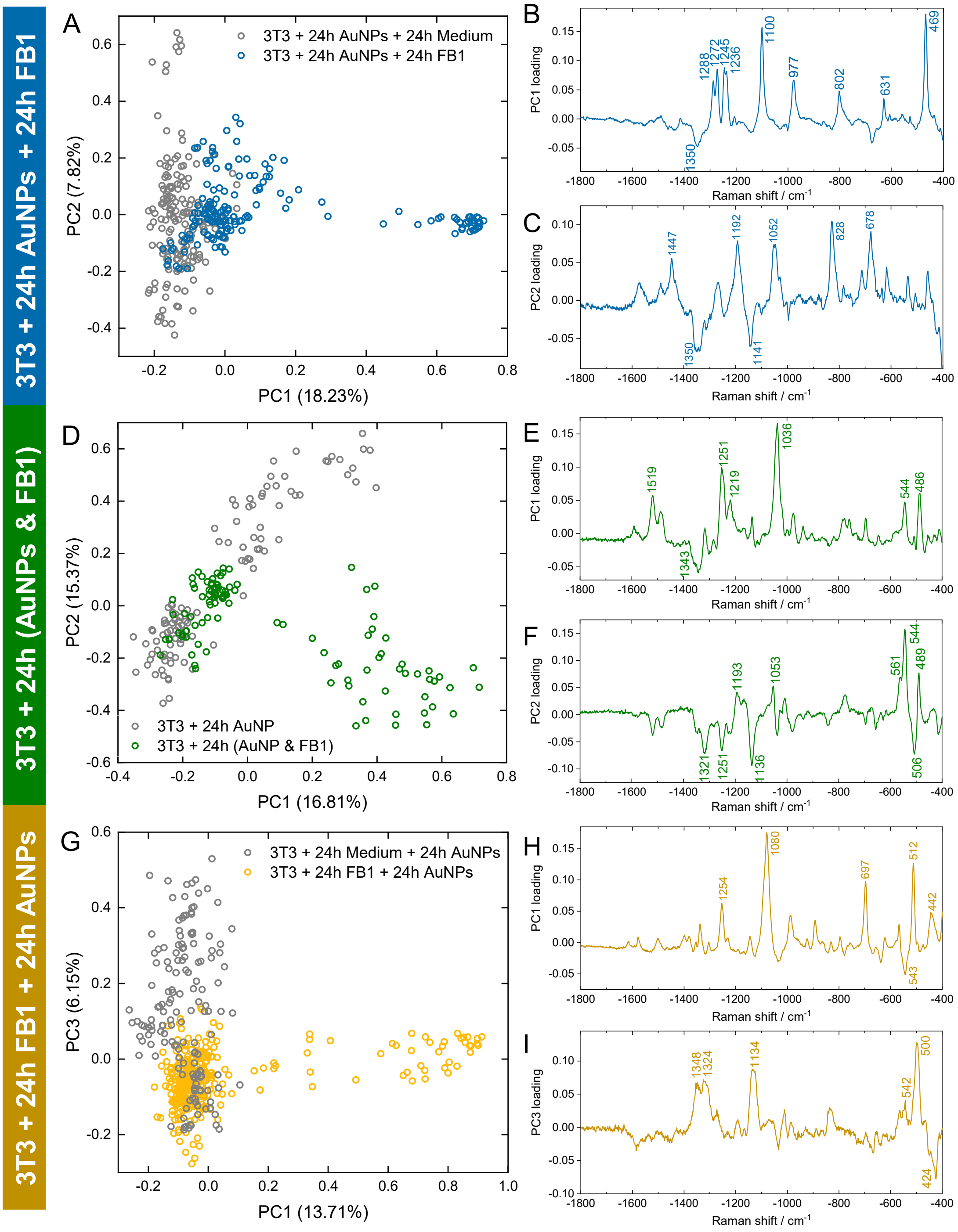

Figure S2. Results of principal component analyses (PCA) applied to data sets of **(A, B, C)** 3T3 cells incubated with gold nanoparticles for 24h prior to 24h incubation with 10 µM FB1 and their control, **(D, E, F)** 3T3 cells incubated with gold nanoparticles together with 10 µM FB1 for 24h and their control, **(G, H, I)** 3T3 cells incubated with gold nanoparticles for 24h after seeding and growing in 10 µM FB1 for 24h and their control groups. The analysis used the full spectral range from 400 cm^{-1} -

1800 cm^{-1}. Score plots of the first two principal components PC1 and PC2 (A, D and G) and the loadings of PC1 and PC2 (B, C, E, F, H and I) are presented.

The SERS spectral variation associated with FB1 treatments exhibited in the loadings of the principal components (Figure S2B, C, E, F, H and J). Different PC1 loadings (Figure S2B, S2E and S2H) indicate that the sequence of FB1 addition relative to the SERS probes affects the endolysosomal environment differently.

Incubation corresponding to Scheme 1A. When cells were exposed to FB1 following gold nanoparticles (Scheme 1A in the main text), the predominant distinctions shown in the PC1 loading (Figure S2B) were similar to the observed differences in the spectra (Figure 1A and 1D), e.g., diverse amide III signals in 1236-1288 cm^{-1}[1, 3-4, 6-7] and different amino acid exposure: tryptophan at 1350 cm^{-1},[3, 6, 12] proline/valine at 977 cm^{-1},[3] tyrosine at 631 cm^{-1}.[7, 12, 15] Variations in lipid signals mainly contributed to the changes due to lipid chain disorder (1100 cm^{-1})[8-9] and a phosphate head group vibration (802 cm^{-1}).[6] The loading of PC2 (Figure S2C) revealed more protein structural differences from the backbone vibration at 1141 cm^{-1},[1, 3] a side chain signal at 1192 cm^{-1}[4-5] and the C-S band at 678 cm^{-1}.[3, 5]

Incubation corresponding to Scheme 1B. As shown by the loading of PC1 (Figure S2E), the most important variance under the simultaneous treatment with FB1 is found in protein structure changes, that include amide II (1519 cm^{-1}),[5] amide III (1251 cm^{-1} and 1219 cm^{-1}),[4, 7] tryptophan (1343 cm^{-1}),[3, 6, 12] tyrosine (1036 cm^{-1})[6] and disulfide group (544 $cm^{-1}$and 489 cm^{-1})[4, 19] signals. The impact from lipid changes was only revealed by bands at 1321 cm^{-1}[3, 13] and 1053 cm^{-1},[8-10] assigned to CH_2/CH_3 deformation and C–C stretching modes, in the loading of PC2 (Figure S2F). These features indicate that the co-treatment must affect protein structure integrity more than the lipid profile in the endolysosomes.

Incubation corresponding to Scheme 1C. When FB1 was introduced prior to SERS nanoprobes (Scheme 1C in the main text), the variation in PC1 (Figure S2H) was dominated by various disulfide bands (442, 512 cm^{-1} and 697 cm^{-1})[1, 3, 5-6, 16] as well as the amide III (1254 cm^{-1})[4, 7] and lipid C–C stretching mode (1080 cm^{-1}),[8-9] in agreement with the observed changes (Figure 1C and 1F). The contributions from tryptophan (1348 cm^{-1}[3, 6, 12] and 424 cm^{-1}[1, 3, 5]), protein C–C/C–N bond (1134 cm^{-1})[1, 3] and lipid signals (1324 cm^{-1})[3, 13] on PC3 (Figure S2I) further imply that protein stability and

membrane structural order may be highly influenced in the endolysosomes of cells with disrupted lipid metabolism in the ER.

Interestingly, under the FB1-subsequent treatment (Scheme 1A in the main text), the PC1 loading was dominated by shared features and exclusive ones from control samples (Figure S2B), different from those of the other two conditions (Figure S2E and S2H) that mainly included the specified spectral features from the treated cells. This suggests that endolysosomes are be more sensitive to effects of decreased ceramide when CerS inhibition in the ER occurs before or simultaneously with the formation of endolysosomal structures around the SERS probes.

Variable importance analysis by surrogate minimal depth from random forest models of SERS data sets of the endolysosomes in 3T3 cells undergoing inhibition of CerS by FB1

Table S2. Performance of random forest analysis to discriminate the spectra of 3T3 cells in different incubation schemes with the inhibitor Fumonisin B1 (FB1) and SERS nanoprobes from their control groups (cf. Scheme 1 in the main text)

**Incubation scheme**	**Classification Accuracy**	
	**Training**	**Test**
(1) 24h nanoprobes<br>(2) 24h FB1	95.36%	91.3%
24h (nanoprobes and FB1)	95.81%	92.86%
(1) 24h FB1<br>(2) nanoprobes	92.71%	96.97%

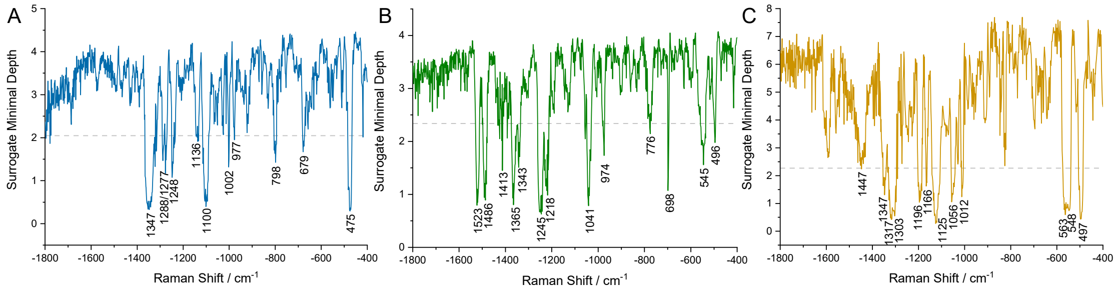

Figure S3. The importance parameter surrogate minimal depth from random forest models of SERS data sets obtained from **(A)** 3T3 cells incubated with gold nanoparticles for 24h prior to 24h incubation with 10 µM FB1 and their control following Scheme 1A in the main text, **(B)** 3T3 cells incubated with gold nanoparticles together with 10 µM FB1 for 24h and their control following Scheme 1B in the main text, **(C)** 3T3 cells incubated with gold nanoparticles for 24h after seeding and growing in 10 µM FB1 for 24h and their control groups following Scheme 1C in the main text. Spectral variables are selected as important bands if they have importance values below the respective threshold, which is shown as a dashed line in each plot. The bands selected as important bands are shown on the vertical axis of the plots of Figure 2 in the main text.

Each of the three separate random forest models, corresponding to the time point of probing vs onset of CerS inhibition (following Scheme 1A-1C), was established using 80% of the spectra as training sets and tested by the remaining 20%. As Table S2 shows, all three models yielded good classification accuracy greater than 90% in both training and test sets. These accurate classification of random forest (RF) models appear more superior to incomplete separation that was observed in the PCA results (Figure S2A, S2D and S2G).

Based on the reliable classification performance, SMD displayed the important variables contributing to group differentiation under FB1 treatments (Figure S3). In the cells exposed to FB1 after the gold nanoparticles (Figure S3A, following Scheme 1A in the main text), SMD identified nearly the same key vibrational features as loadings of PC1 and PC2 (Figure S2B and S2C), with an additional band at 1002 cm^{-1} assigned to the phenylalanine ring breathing mode.[4, 12]

When FB1 was added into cells together with the gold nanoparticles (following Scheme 1B in the main text), more different important bands were identified by SMD (Figure S3B) than were found in the PCA loadings (Figure S2E and S2F). They include contributions by amide II at 1486 cm^{-1},[5] tryptophan vibrations at 1413 cm^{-1},[18] 1365 cm^{-14} and 776 cm^{-1},[4-5, 21] a proline/valine band at 974 cm^{-1},[4] and a C-S stretching band at 698 cm^{-17} (Figure S3B). Increased contributors from protein, especially various tryptophan vibrations, coupled with fewer lipid features pointed to protein conformational changes as the predominant distinction in the classification between co-treated cells and control samples in the RF model.

Upon the administration of FB1 prior to gold nanoparticles (following Scheme 1C in the main text), SMD revealed several more extensive lipid signatures that differentiate treated cells from the control group, such as a methylene scissoring mode at 1447 cm^{-1},[1, 3-4, 6] a CH_2 deformation at 1303 cm^{-1},[6-7, 15] intra-chain C–C stretching vibrations at 1125 cm$^{-14, 6, 8}$ and 1056 cm^{-18-10} (Figure S3C). Moreover, signals from aromatic residues, tyrosine/tryptophan/phenylalanine at 1196 cm^{-1},[4-5] phenylalanine at 1012 cm^{-1},[4] and tryptophan at 563 cm^{-14-5} (Figure S3C), contributed to the spectral differences induced by FB1 pre-treatment, as selected by RF-SMD. The amide III band (1254 cm^{-1})[4, 7] sensitive to protein secondary structure in the PCA loadings (Figure S2G) is absent here.

Ultrastructure of 3T3 cells incubated with gold nanoparticles as control samples

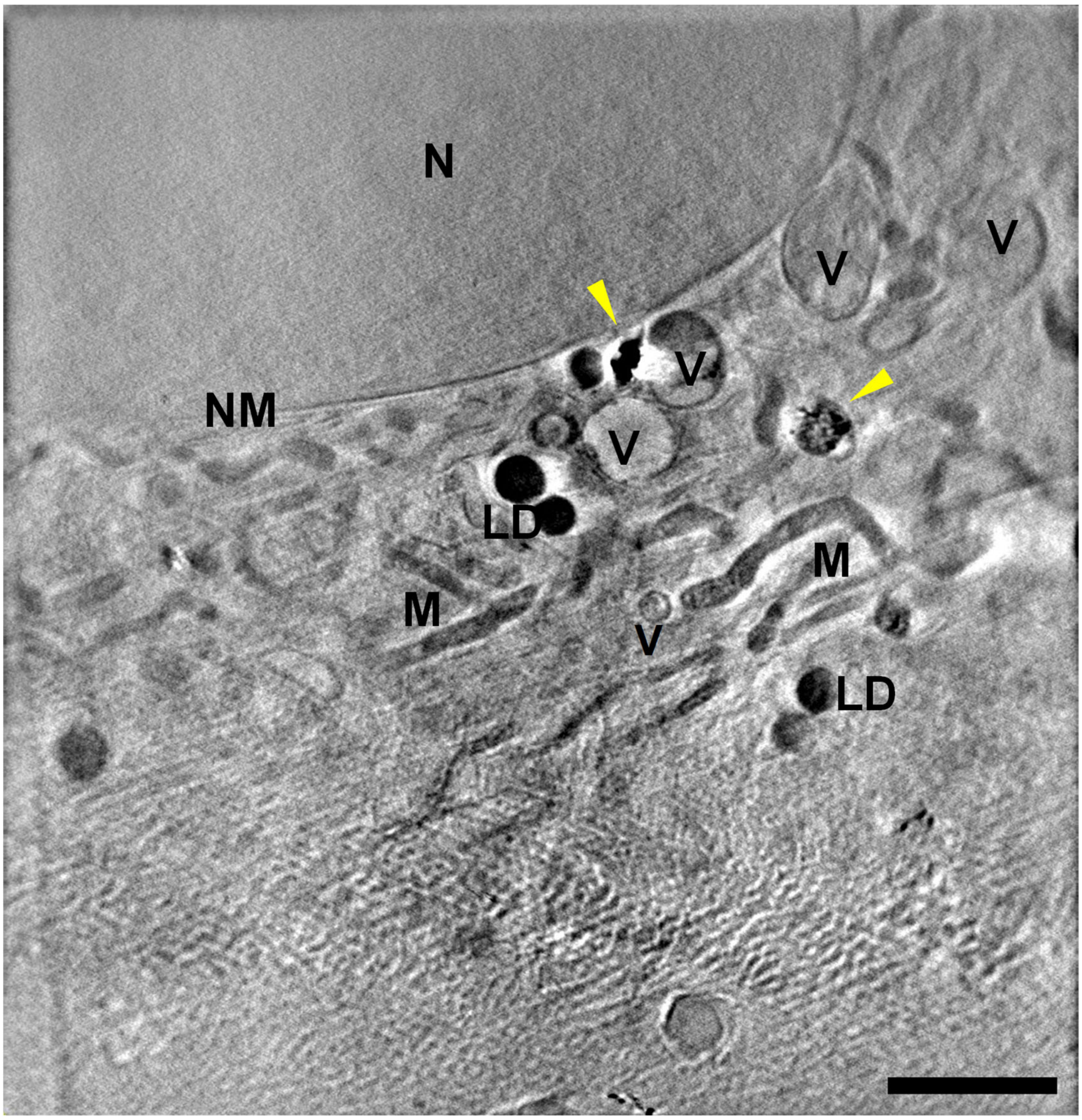

Figure S4. Tomographic slices of 3T3 cells incubated with gold nanoparticles for 24h as control samples. M: mitochondrion, V: vesicle, NM: nuclear membrane, N: nucleus, LD: lipid droplets. Gold nanostructures are marked with yellow arrowheads. Scale bar: 2 μm.

Methods

Preparation of cell samples

Swiss albino mouse fibroblast cell line 3T3 (DSMZ, Braunschweig, Germany) were cultured independently in Dulbecco's Modified Eagle Medium (DMEM, Bio&SELL, Nürnberg, Germany) supplemented with 10% fetal calf serum (FCS, Biochrom, Berlin, Germany) under standard conditions (37°C, 5% CO_2).

Preparation of cells for SERS experiments. For the SERS experiment, 3T3 cells were seeded on glass cover slips at a density of 4000-8000 cells per slip. The citrated-stabled gold nanoparticles as SERS nanoprobes were synthesized by the protocol described by Lee and Meisel.[23]

For SERS experiments of FB1 treatment after nanoprobe incubation (following Scheme 1A in the main text), after cells were grown on glass cover slips for 24h, the culture medium was exchanged with a dilution of gold nanoparticles in DMEM-FCS of 1:10 (nanoparticle concentration ~10^{-11} M) for 24h. The cells were rinsed with phosphate-buffered saline (PBS, Bio&SELL, Nürnberg, Germany) to remove excess gold nanoparticles before any treatments. Then, the cells were incubated at 37°C in DMEM-FCS with 10 μM FB1 for 24h. For co-treatment with FB1 and AuNPs (following Scheme 1B in the main text), after cells were grown on glass cover slips for 24h, cells were incubated in 10 μM FB1 and gold nanoparticles diluted in 1:10 (nanoparticle concentration ~10^{-11} M) in DMEM-DCS for 24h. Control samples were incubated only with gold nanoparticles. For incubation pre-treated with FB1 (following Scheme 1C in the main text), cells were grown with 10 μM FB1 in DMEM-FCS at 37°C for 24h. After cells were rinsed by PBS, the culture medium was exchanged with a dilution of gold nanoparticles in DMEM-FCS of 1:10 (nanoparticle concentration ~10^{-11} M) for 24h. SERS spectra from the cells were measured in PBS after rinsing with PBS three times.

Preparation of cells for cryo soft X-ray nanotomography. For cryo soft X-ray nanotomography (SXT), 3T3 cells were grown on Formvar-coated gold grids (Quantifoil, Jena, Germany) and incubated as described above. After the incubation, each grid was rinsed three times with PBS, the excess of buffer was blotted with a filter paper, and the grids were plunge-frozen in liquid ethane.

SERS experiments

All Raman spectra were excited by a 785 nm diode laser (Toptica, Munich, Germany). SERS spectra were obtained using an intensity of 2.7×10^5 W cm^{-2} yielded when focusing the excitation light with a 60× water immersion objective into cellular samples. Raman light was directed to a single-stage

spectrograph equipped with a liquid nitrogen-cooled CCD detector (Horiba, Munich, Germany). The spectral resolution is ~2 cm^{-1} considering the full spectral range of 300-1900 cm^{-1}. From each sample, spectra were collected using an acquisition time of 1 s per spectrum.

Cryogenic soft X-ray tomography of cell samples

Vitrified cell monolayers with a thickness of approximately 10 µm were examined using a transmission X-ray microscope equipped with a cryostage at beamline U41-PGM1-XM at the electron storage ring BESSY II (Helmholtz-Zentrum Berlin für Materialien und Energie, Berlin, Germany)[24]. Tilt series of individual cells were acquired at different angular ranges in increments of 1° at an image pixel size of 9.8 nm (25 nm zone plate objective) and a photon energy of 510 eV. Depending on the sample thickness, the exposure time was adjusted for each tilt angle series between 1 s and 3 s per image. The tilt series consist of up to 130 images. Tomograms were obtained by alignment of the corrected tilt series using the intracellular gold nanoparticles as fiducial markers and tomographic reconstruction using the Etomo software (IMOD, Colorado, USA). The reconstruction of sample volumes was carried out by back-projection or simultaneous iterative reconstruction technique.

Data analysis

Preprocessing of SERS spectra. The SERS spectra were frequency-calibrated using a spectrum of a toluene−acetonitrile mixture (1:1) and preprocessed regarding removal of spikes, baseline correction using asymmetric least squares (AsLS)[25], and vector-normalization in MatLab R2020b (The MathWorks, Inc., Natick, MA, USA). After removal of spectra with no signal from the data sets, the remaining spectra in a range of 400-1800 cm^{-1} were subjected to different analyses. The relative occurrence of signals in individual spectra with background correction and vector normalization were analyzed using a script in Mathematica 12.1 (Wolfram, Champaign, IL, USA) as detailed in ref. [1].

Principal component analysis. Principal component analysis (PCA) was performed within MATLAB R2020b (The MathWorks, Inc., Natick, MA, USA) using the 'pca' function on mean-centered SERS spectra with signals.

Random Forest based surrogate minimal depth. SMD is based on surrogate variables, which have been introduced to enable the analysis of datasets containing missing values with random forest.[26] In SMD, however, surrogate variables are used to incorporate variable relationships into importance analyses and assess the mutual impact of variables on the random forest model. For the latter, the adjusted agreement is exploited. This parameter quantifies the similarity between the original split and potential surrogate splits at each node in the decision trees, in order to find appropriate surrogate

variables. A simulation study has demonstrated the applicability of SMD to SERS data, thereby facilitating the identification of co-occurring SERS signals, even when these signals are present in a limited number of spectra.[27]

RF-based SMD analyses were conducted in R 4.2.2[28] using *ranger* 0.15.1[29] and *RFSurrogates* 0.4.2[30-31], respectively. For each comparison, an RF classification model was trained to differentiate between the spectra of the respective condition and the control samples. This model was trained using 80% of the spectra that were randomly selected and the remaining 20% of the spectra were used as test data. For training, the number of trees (*num.trees*) and the number of variables to consider in each split (*mtry*) were set to 10'000 and 161 (corresponding to $p^{3/4}$ where p is the total number of spectral variables), respectively. The classification accuracy for the training and test data was determined by calculating the ratio of correct predictions to the total number of respective SERS spectra. SMD was performed using the RF parameters defined above and a predefined number of surrogate splits (s) of 88, corresponding to 0.10 p. Important variables were selected based on the SMD importance score with lower value indicating greater importance. Consequently, variables with SMD values below the defined threshold were selected and the mean adjusted agreement (MAA) for pairwise relationships of each spectral variable was determined. For clarity, the variable with the lowest SMD importance value served as representative for the signals corresponding to one signal. Their MAA to all p spectral variables were depicted to visualize the relation between the important signals and spectral variables characteristic of specific molecules, which can reflect the co-localization of molecular groups adjacent to nanoparticles.

References

(1) Szekeres, G. P.; Montes-Bayón, M.; Bettmer, J.; Kneipp, J., Fragmentation of Proteins in the Corona of Gold Nanoparticles as Observed in Live Cell Surface-Enhanced Raman Scattering. *Anal. Chem.* **2020,** *92*, 8553-8560.
(2) Rygula, A.; Majzner, K.; Marzec, K. M.; Kaczor, A.; Pilarczyk, M.; Baranska, M., Raman Spectroscopy of Proteins: A Review. *J. Raman Spectrosc.* **2013,** *44*, 1061-1076.
(3) Spedalieri, C.; Szekeres, G. P.; Werner, S.; Guttmann, P.; Kneipp, J., Intracellular Optical Probing with Gold Nanostars. *Nanoscale* **2021,** *13*, 968-979.
(4) Movasaghi, Z.; Rehman, S.; Rehman, I. U., Raman Spectroscopy of Biological Tissues. *Appl. Spectrosc. Rev.* **2007,** *42*, 493-541.
(5) Szekeres, G. P.; Werner, S.; Guttmann, P.; Spedalieri, C.; Drescher, D.; Živanović, V.; Montes-Bayón, M.; Bettmer, J.; Kneipp, J., Relating the Composition and Interface Interactions in the Hard Corona of Gold Nanoparticles to the Induced Response Mechanisms in Living Cells. *Nanoscale* **2020,** *12*, 17450-17461.
(6) Živanović, V.; Seifert, S.; Drescher, D.; Schrade, P.; Werner, S.; Guttmann, P.; Szekeres, G. P.; Bachmann, S.; Schneider, G.; Arenz, C.; Kneipp, J., Optical Nanosensing of Lipid Accumulation Due to Enzyme Inhibition in Live Cells. *ACS Nano* **2019,** *13*, 9363-9375.
(7) Pezzotti, G., Raman Spectroscopy in Cell Biology and Microbiology. *J. Raman Spectrosc.* **2021,** *52*, 2348-2443.
(8) Guillard, E.; Tfayli, A.; Manfait, M.; Baillet-Guffroy, A., Thermal Dependence of Raman Descriptors of Ceramides. Part II: Effect of Chains Lengths and Head Group Structures. *Anal. Bioanal. Chem.* **2011,** *399*, 1201-1213.
(9) Tfayli, A.; Guillard, E.; Manfait, M.; Baillet-Guffroy, A., Molecular Interactions of Penetration Enhancers within Ceramides Organization: A Raman Spectroscopy Approach. *Analyst* **2012,** *137*, 5002.
(10) Rothschild, K. J.; Andrew, J. R.; Grip, W. J. D.; Stanley, H. E., Opsin Structure Probed by Raman Spectroscopy of Photoreceptor Membranes. *Science* **1976,** *191*, 1176-1178.
(11) Notingher, I.; Green, C.; Dyer, C.; Perkins, E.; Hopkins, N.; Lindsay, C.; Hench, L. L., Discrimination between Ricin and Sulphur Mustard Toxicity in Vitro Using Raman Spectroscopy. *J. R. Soc. Interface* **2004,** *1*, 79-90.
(12) Tang, S.; Gao, S.; Xu, J.; Zheng, M.; Huang, Y.; Yu, Y.; Lin, J., A Novel Serum Protein Purification Technique Combined with Surface-Enhanced Raman Spectroscopy for Liver Cancer Detection. *Spectrosc. Lett.* **2021,** *54*, 113-121.
(13) Feng, Y.; Gärber, F.; Saied, E. M.; Spedalieri, C.; Kochovski, Z.; Werner, S.; Pratsch, C.; Arenz, C.; Seifert, S.; Kneipp, J., SERS Spectra Indicate the Molecular Effects of 7-Nitrobenz-2-oxa-1,3-diazole (NBD) on Living Cells. *J. Phys. Chem. C* **2024,** *128*, 19722-19735.
(14) Podstawka, E.; Ozaki, Y.; Proniewicz, L. M., Part III: Surface-Enhanced Raman Scattering of Amino Acids and Their Homodipeptide Monolayers Deposited onto Colloidal Gold Surface. *Appl. Spectrosc.* **2005,** *59*, 1516-1526.
(15) Stone, N.; Kendall, C.; Smith, J.; Crow, P.; Barr, H., Raman Spectroscopy for Identification of Epithelial Cancers. *Faraday Discuss.* **2004,** *126*, 141-157.
(16) Lopez-Tobar, E.; Hernández, B. n.; Ghomi, M.; Sanchez-Cortes, S., Stability of the Disulfide Bond in Cystine Adsorbed on Silver and Gold Nanoparticles as Evidenced by SERS Data. *J. Phys. Chem. C* **2013,** *117*, 1531-1537.
(17) Garidel, P.; Folting, B.; Schaller, I.; Kerth, A., The Microstructure of the Stratum Corneum Lipid Barrier: Mid-Infrared Spectroscopic Studies of Hydrated Ceramide:Palmitic Acid:Cholesterol Model Systems. *Biophys. Chem.* **2010,** *150*, 144-56.

(18) Madzharova, F.; Heiner, Z.; Gühlke, M.; Kneipp, J., Surface-Enhanced Hyper-Raman Spectra of Adenine, Guanine, Cytosine, Thymine, and Uracil. *The Journal of Physical Chemistry. C, Nanomaterials and Interfaces* **2016,** *120*, 15415-15423.
(19) Parker, F. S., *Applications of Infrared, Raman, and Resonance Raman Spectroscopy in Biochemistry*. Springer Science & Business Media: 1983.
(20) Feng, Y.; Kochovski, Z.; Arenz, C.; Lu, Y.; Kneipp, J., Structure and Interaction of Ceramide-Containing Liposomes with Gold Nanoparticles as Characterized by SERS and Cryo-EM. *J. Phys. Chem. C* **2022,** *126*, 13237-13246.
(21) Simon, I.; Hedesiu, M.; Virag, P.; Salmon, B.; Tarmure, V.; Baciut, M.; Bran, S.; Jacobs, R.; Falamas, A., Raman Micro-Spectroscopy of Dental Pulp Stem Cells: An Approach to Monitor the Effects of Cone Beam Computed Tomography Low-Dose Ionizing Radiation. *Anal. Lett.* **2019,** *52*, 1097-1111.
(22) Živanović, V.; Milewska, A.; Leosson, K.; Kneipp, J., Molecular Structure and Interactions of Lipids in the Outer Membrane of Living Cells Based on Surface-Enhanced Raman Scattering and Liposome Models. *Anal. Chem.* **2021,** *93*, 10106-10113.
(23) Lee, P.; Meisel, D., Adsorption and Surface-Enhanced Raman of Dyes on Silver and Gold Sols. *J. Phys. Chem.* **1982,** *86*, 3391-3395.
(24) Schneider, G.; Guttmann, P.; Heim, S.; Rehbein, S.; Mueller, F.; Nagashima, K.; Heymann, J. B.; Müller, W. G.; McNally, J. G.; Schneider, G.; Guttmann, P.; Heim, S.; Rehbein, S.; Mueller, F.; Nagashima, K.; Heymann, J. B.; Müller, W. G.; McNally, J. G., Three-Dimensional Cellular Ultrastructure Resolved by X-Ray Microscopy. *Nat. Methods* **2010,** *7*, 985-987.
(25) Eilers, P. H., A Perfect Smoother. *Anal. Chem.* **2003,** *75*, 3631-3636.
(26) Breiman, L.; Friedman, J.; Stone, C.; Olshen, R., *Classification and Regression Trees*. Taylor & Francis: 1984.
(27) Seifert, S., Application of Random Forest Based Approaches to Surface-Enhanced Raman Scattering Data. *Sci. Rep.* **2020,** *10*, 5436.
(28) Team, R. C. *R: A Language and Environment for Statistical Computing*; R Foundation for Statistical Computing: Vienna, Austria, 2022, 2022.
(29) Wright, M. N.; Ziegler, A., Ranger: A Fast Implementation of Random Forests for High Dimensional Data in C++ and R. *J. Stat. Softw.* **2017,** *77*, 5753-5771.
(30) Voges, L. F.; Jarren, L. C.; Seifert, S., Exploitation of Surrogate Variables in Random Forests for Unbiased Analysis of Mutual Impact and Importance of Features. *Bioinformatics* **2023,** *39*, btad471.
(31) Seifert, S.; Gundlach, S.; Szymczak, S., Surrogate Minimal Depth as an Importance Measure for Variables in Random Forests. *Bioinformatics* **2019,** *35*, 3663-3671.
